# Sacramento Medical Society

**Published:** 1856-04

**Authors:** 


*Sacramento Medical Society*—We have received the Preamble and Resolutions, looking to the organization of a Medical Society in California. We are pleased that our friends of the *auriferous* appendix to our republic, find other subjects of investigation and thought, than the acquirement of the "root of all evil." Already, if we mistake not, they have set on foot the formation of a State Society there also, from which we should be pleased to hear thoroughly the proceedings.

At a meeting of the Faculty of the College of "Physicians and Surgeons of the Iowa University," Professors M'Gugin and Hughes were appointed delegates to the "American Medical Association," to convene at Detroit, Michigan. 1856

